# Spatial patterns of Bovine Corona Virus and Bovine Respiratory Syncytial Virus in the Swedish beef cattle population

**DOI:** 10.1186/1751-0147-52-33

**Published:** 2010-05-21

**Authors:** Francois Beaudeau, Camilla Björkman, Stefan Alenius, Jenny Frössling

**Affiliations:** 1 ONIRIS, UMR 1300 Bioagression, Epidémiologie et Analyse de Risque, BP 40706, F-44307, Nantes, France; 2 INRA, UMR 1300 Bioagression, Epidémiologie et Analyse de Risque, BP 40706, F-44307, Nantes, France; 3 Université Nantes Angers Le Mans, Nantes, France; 4 Department of Clinical Sciences, Swedish University of Agricultural Sciences, PO Box 7054, SE-750 07, Uppsala, Sweden; 5 Department of Disease Control and Epidemiology, National Veterinary Institute (SVA), SE-751 89 Uppsala, Sweden

## Abstract

**Background:**

Both bovine coronavirus (BCV) and bovine respiratory syncytial virus (BRSV) infections are currently wide-spread in the Swedish dairy cattle population. Surveys of antibody levels in bulk tank milk have shown very high nationwide prevalences of both BCV and BRSV, with large variations between regions. In the Swedish beef cattle population however, no investigations have yet been performed regarding the prevalence and geographical distribution of BCV and BRSV. A cross-sectional serological survey for BCV and BRSV was carried out in Swedish beef cattle to explore any geographical patterns of these infections.

**Methods:**

Blood samples were collected from 2,763 animals located in 2,137 herds and analyzed for presence of antibodies to BCV and BRSV. Moran's *I *was calculated to assess spatial autocorrelation, and identification of geographical cluster was performed using spatial scan statistics.

**Results:**

Animals detected positive to BCV or BRSV were predominately located in the central-western and some southern parts of Sweden. Moran's *I *indicated global spatial autocorrelation. BCV and BRSV appeared to be spatially related: two areas in southern Sweden (Skaraborg and Skåne) had a significantly higher prevalence of BCV (72.5 and 65.5% respectively); almost the same two areas were identified as being high-prevalence clusters for BRSV (69.2 and 66.8% respectively). An area in south-east Sweden (Kronoberg-Blekinge) had lower prevalences for both infections than expected (23.8 and 20.7% for BCV and BRSV respectively). Another area in middle-west Sweden (Värmland-Dalarna) had also a lower prevalence for BRSV (7.9%). Areas with beef herd density > 10 per 100 km^2 ^were found to be at significantly higher risk of being part of high-prevalence clusters.

**Conclusion:**

These results form a basis for further investigations of between-herds dynamics and risk factors for these infections in order to design effective control strategies.

## Background

Bovine coronavirus (BCV) and bovine respiratory syncytial virus (BRSV) are frequently involved in the respiratory and enteric disease complexes of cattle [1,2]. BCV is causing winter dysentery in adults [3,4], calf diarrhoea [5] and also respiratory disease of young stock [6]. BRSV is recognized as one of the most important causes of respiratory tract disease in beef and dairy cattle, especially in young animals [7-9].

Presence of antibodies to BCV [10-15] and to BRSV [9,10,12,16,17] has been reported worldwide in both dairy and beef cattle.

Both BCV infection and BRSV infection are considered relatively contagious and are currently wide-spread in the Swedish dairy cattle population. Surveys of antibody levels in bulk tank milk have shown very high nationwide prevalences of both BCV [13] and BRSV [9], with large variations between regions. The highest herd-prevalences (90 to 100%) were found in the southern parts of the country. It was suggested that a reason for the higher BCV prevalence in the south could be the high dairy-herd density, associated with an increased risk of spread between herds through infected animals, vectors and airborne transmission [13]. In the Swedish beef cattle population however, no investigations have yet been performed regarding the prevalence and geographical distribution of BCV and BRSV.

The aim of the present study was to identify possible high risk areas for BCV and BRSV infections in the beef cattle population in Sweden, and further to explore whether a high beef herd-density was a risk factor for higher seroprevalences.

## Materials and methods

### Study design

The cross-sectional study was conducted on blood samples collected within the Swedish Bovine Viral Diarrhoea (BVD) surveillance program. Within this program, all Swedish herds are required to be tested on a regular basis to maintain their BVD free status [18]. For beef cattle herds, depending on the number of dams present in the herd, five to ten blood samples are taken in young stock over 12 months of age per herd-year and sent to the National Veterinary Institute where they are analyzed for presence of BVDV antibodies [19]. In total, approximately 45,000 blood samples are collected annually from beef herds.

Between November 2006 and May 2007, every 12^th ^blood sample was systematically selected for an investigation of *Neospora caninum *in Swedish beef cattle [20]. The same study sample was used here; it consisted of 2,763 serum samples originating from 2,137 herds, corresponding to approximately 20% of all beef herds present in the country at this time. The sample was considered to be representative of the Swedish beef cattle population, as it was issued from a procedure functionally similar to a random sampling. The number of blood samples taken per herd ranged from 1 to 8, but most herds were represented by one or two samples (81 and 14%, respectively).

### Diagnostic tests

The samples were analysed for presence of immunoglobulin G antibodies to BCV [4] and BRSV [21] by commercially available indirect enzyme-linked immunosorbent assays (ELISA; SVANOVA Biotech, Uppsala, Sweden). The optical density (OD) at 450 nm was corrected by subtraction of the negative control antigen OD. Cut-off was set to a corrected OD of 0.20, which is recommended by the manufacturer for individual samples. At this cut-off, the sensitivity is estimated to 84.6% for BCV and 94.6% for BRSV and specificity to 100% for both tests (SVANOVA manual). A sample was considered test positive if its corrected OD was >0.20, and test negative otherwise.

### Location data

The locations of all Swedish beef herds, including the herds where the blood samples were collected, were specified by three-digit postal codes. Postal codes were retrieved from the database of the organization responsible for the BVD surveillance program, i.e. the Swedish Dairy Association (year 2007). Applicable postal codes were available for 2,757 samples from 2,131 beef herds in the study population.

### Spatial analyses

For BCV and BRSV infections separately, the spatial distribution of samples and possible clustering were investigated by following the same procedure using data aggregated by postal code area (PCA).

The crude prevalence was defined as the number of positive samples over the total number of samples and was calculated for each PCA. This raw prevalence was adjusted by applying a Spatial Empirical Bayes smoothing (SEB), i.e. adjusted (i) for the potential biasing effects of variance instability due to differences in the size of the population at risk, and (ii) considering the estimates from neighboring areas [22]. Presence of global spatial autocorrelation was tested using the Moran's *I *test for SEB rates [23]. Its significance was calculated by Monte-Carlo simulation. All smoothing and testing for spatial associations of area aggregated data was performed using the GeoDa software version 0.9.5-i5 http://geodacenter.asu.edu/.

Identification of potential clusters of positive samples was based on location determined by PCA centroids, and using the spatial scan statistic (M. Kulldorff and Information Management Services, Inc. SatScan version 8.0, http://www.satscan.org, 2009). The method is based on either circles or ellipses centered on each PCA centroid; a Relative Risk can be estimated which compares the risk of being a case inside the circle/ellipse to the risk of being a case outside the circle/ellipse [24]. A circle/ellipse is considered a cluster if the Relative Risk is significantly higher or lower than one, when significance was tested using Monte-Carlo simulation. In this study, Poisson models applying both different cluster shapes (circular and elliptic) and sizes (maximum cluster sizes of 50, 20 and 10% of the total population at risk) were built to identify both high-risk and low-risk clusters. No overlapping of the circles/ellipses was allowed.

BCV and BRSV are contagious, and test positive animals are expected to be grouped in herds. As the likelihood of detecting at least one test positive animal increases with the number of individual samples collected per herd, identification of clusters and their spatial location might be biased if the herds from which two or more blood samples (19% of the studied herds) were collected are not evenly geographically-distributed. To investigate this potential bias in relation to the sampling strategy, possible clustering of herds with more than one sampled animal was explored in a preliminary step by using Moran's *I*. This test indicated that these herds were proportionally distributed over Sweden (data not shown).

To test whether or not a high beef herd-density was a risk factor for significantly higher seroprevalences of BCV and BRSV than expected, univariate logistic regression analysis was performed at PCA level, where the binary outcome was "PCA with a significantly higher number of positive-tested samples *vs. *without" and the putative explanatory variable was the PCA herd-density (in herds/100 km^2^) in 3 classes [<5; 5-10; >10].

Data management, statistics and creation of map shape-files were performed using SAS 9.2 (SAS Institute, Inc., Cary, NC, USA) and ArcGIS 9.1 (ESRI Inc., Redlands, CA, USA).

## Results

**Figure 1:**
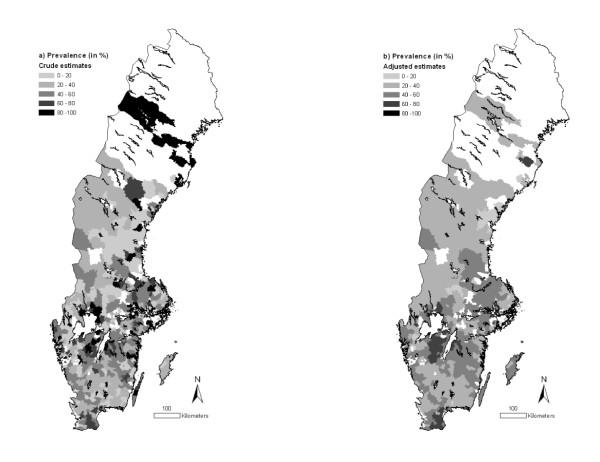
**Prevalence of BCV in Swedish beef cattle by three-digit postal code area (2007)**. The estimates presented are (a) crude or (b) adjusted by empirical Bayes smoothing applying a spatial weight matrix. Information was missing for the white areas. ^© ^Lantmäteriverket Gävle 2010. Permission number I 2010/0055.

**Figure 2:**
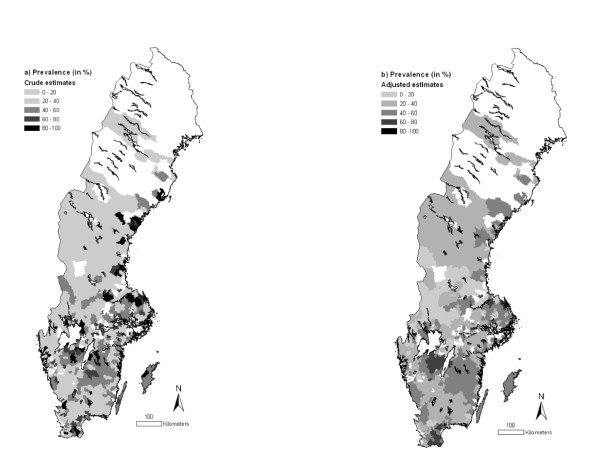
**Prevalence of BRSV in Swedish beef cattle by three-digit postal code area (2007)**. The estimates are (a) crude or (b) adjusted by empirical Bayes smoothing applying a spatial weight matrix. Information was missing for the white areas. ^© ^Lantmäteriverket Gävle 2010. Permission number I 2010/0055.

The overall prevalence of animals testing positive to BCV and BRSV was 43.1 (95% CI: 41.3-45.0) and 39.2% (95% CI: 37.3-41.0) respectively. There was a statistically significant (P < 0.01, χ^2^-test) relationship between BCV and BRSV serological status, i.e. BCV-positive animals were more likely to be BRSV-positive and *vice versa*. Animals testing positive to BCV were predominately located in the central-western and southern Sweden, as well as in some northern areas (Figure 1a). Animals testing positive to BRSV were predominately found in the same central-western and southern parts of the country (Figure 2a). When the prevalences were adjusted by SEB, these tendencies became even clearer (Figures 1b and 2b) and the northern areas were no longer considered having high prevalences for BCV.

The findings were confirmed by significant Moran's *I *tests for both infections (0.15, P = 0.0001 for BCV; 0.16, P = 0.001 for BRSV), suggesting that the test positive animals were not randomly distributed throughout the country.

**Figure 3:**
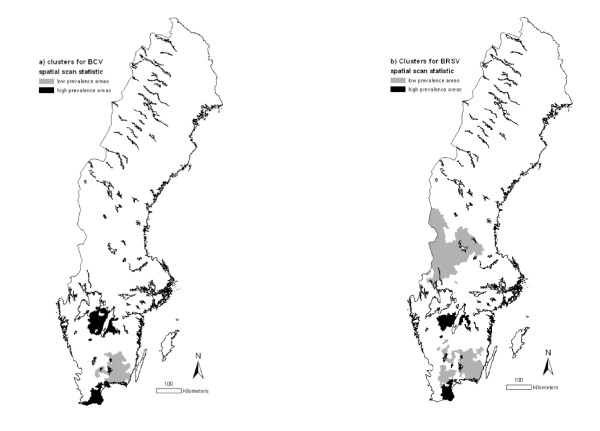
**Areas with high or low prevalences of (a) BCV and (b) BRSV, obtained by a spatial scan statistic (Kulldorff, 1997), using the centroids of the three-digit postal code areas as coordinates (p < 0.01)**. ^© ^Lantmäteriverket Gävle 2010 Permission number I 2010/0055.

Using the spatial scan statistic with elliptic clusters and a maximum cluster size of 10% of the population at risk identified two areas with higher prevalence of BCV than expected: Skaraborg (central-south part of Sweden) and Skåne (extreme south), as well as one area with lower prevalence than expected: Kronoberg-Blekinge (south-east of Sweden; Figure 3a). Almost the same high prevalence areas (Skaraborg and Skåne) were identified as being clusters for BRSV. Two areas with low prevalence of BRSV were also detected: Kronoberg-Blekinge and Värmland-Dalarna (middle-west) (Figure 3b). The characteristics of the identified clusters are displayed in Table 1.

**Table 1:** Characteristics of the three areas in Sweden with significantly higher or lower BCV and BRSV prevalences obtained by a spatial scan statistic (Kulldorff, 1997).

Clusters	BCV				BRSV			
		
	**Area (km**^**2**^**)**	Samples (n)	Prevalence (%)	**RR**^**1**^	**Area (km**^**2**^**)**	Samples (n)	Prevalence (%)	**RR**^**1**^
Skaraborg	7,585	207	72.5	1.77**	4,734	156	69.2	1.85**
Skåne	3,748	254	65.5	1.51**	2,852	223	66.8	1.82**
Kronoberg-Blekinge	10,145	247	23.8	0.53**	15,397	401	20.7	0.51*
Värmland-Dalarna	-		-	-	31,435	89	7.9	0.20**

^1 ^Relative Risk

* < 0.05

** < 0.001

**Figure 4:**
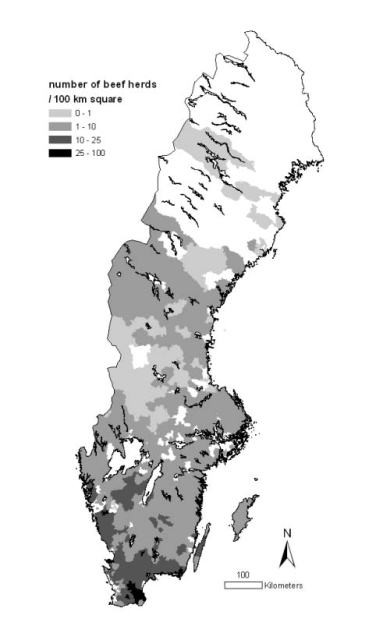
**The population of Swedish beef herds presented as density by three-digit postal code area (2007)**. ^© ^Lantmäteriverket Gävle 2010. Permission number I 2010/0055.

The beef-herd density per PCA is presented in Figure 4. Among the 34 PCAs included in the clusters of high BCV prevalence, 20 had a beef herd-density >10 herds/100 km^2^. This proportion was 14 out of 22 for BRSV. For BCV, the risk for a PCA to be part of a cluster of high prevalence was 5.3 times (95% CI: 2.5-11.1) higher if its beef herd-density was >10 herds/100 km^2 ^than if it was <10 herds/100 km^2^; this risk was 6.1 times (95% CI: 2.4-15.1) higher for BRSV.

## Discussion

This study showed, from visual inspection and descriptive analyses, that both BCV and BRSV infections are very frequent in Swedish beef cattle, especially in some central-western and southern parts of the country. The results are in accordance with what has been reported for dairy cattle where prevalences of BCV- and BRSV-infected dairy herds have been shown to increase with a gradient southward in the country [9,13,25]. A recent study has also shown substantial production effects (e.g. reduced milk yield) associated with BRSV infection in Swedish dairy herds [26].

There was a strong relationship at animal level between being tested positive to BCV and BRSV, and exploratory spatial analyses also indicated two areas with particularly high prevalences for both BCV and BRSV, i.e. Skaraborg and Skåne. The association between BCV and BRSV is biologically plausible because both viruses are relatively contagious and have transmission routes that are to some extent similar. A concomitant BRSV and BCV infection burden and spread in calves has also been demonstrated [10,27].

For both infections, the prevalence of animals testing positive was approximately 40%. However these apparent prevalences at individual level are probably underestimated. Because a few individuals were sampled from each herd (one or two in 95% of herds in the study sample) the herd sensitivity was here low. As a consequence, it can be assumed that a proportion of infected herds and of infected individuals within these herds could have been missed (as both viruses are highly contagious within herd), thus decreasing the apparent individual-level prevalences. This study was not predominately designed to estimate the seroprevalences of BCV and BRSV infections in Sweden, but to explore their spatial distribution.

Both high prevalence areas have a relatively high density of beef herds (13 and 22 per 100 km^2 ^in Skaraborg and Skåne, respectively). In addition, the area with the lowest prevalence for BRSV has a very low beef herd density (2 per 100 km^2^). This suggests a positive association between herd-density and risk of infection. Statistical analysis confirmed that areas with herd-density >10 per 100 km^2 ^had significantly higher risk of being part of high-prevalence clusters. A probable explanation is that a short distance between herds increases the risk of spread of the viruses. In such situations, there is a higher likelihood of direct and indirect contact (through e.g. animals, vehicles or visitors) between herds and animals. However, some PCAs in the low-prevalence cluster Kronoberg-Blekinge also had a beef herd-density >10 per 100 km^2^, suggesting that there are other factors with uneven geographic distribution that have an impact on the BCV and BRSV prevalences. Live animal trade, in particular, is considered very important for the spread of infectious diseases and a recent study show that the number of movements and trade patterns in different parts of Sweden vary considerably [28].

Large herd size has also been identified as risk factor for BCV and BRSV infections in dairy cattle [13,27] and for respiratory disease outbreaks in beef cattle [29]: on increasing the herd size from 20 to 50 animals, the risk for disease outbreak increased 2.1-fold. The size of Swedish beef herds differs between regions and there is a tendency for smaller herd sizes in the regions covering the identified low prevalence areas compared to the high prevalence areas (10 beef adults per herd in Kronoberg-Blekinge versus 16 in Skåne, based on information from the database of the Swedish Board of Agriculture). Also regional differences in biosecurity and management routines in relation to farming styles can be assumed. Based on a study on Swedish dairy farms, it has been suggested that organic herds may have a reduced risk of BCV and BRSV infections, when compared to conventional herds [30]. To quantify the relative impact of potential risk factors for a herd to be detected as BCV or BRSV-infected (e.g. trade intensity, density of dairy herds, herd size, biosecurity measures, type and number of visitors), studies could be conducted by comparing herd characteristics and management practices in the low and high-risk areas identified. The results of such studies would enlighten the choice of relevant strategies to control BCV and BRSV infections not only in Sweden, but also in other areas where herds have to some extent similar characteristics.

## Conclusions

The present study shows that BCV and BRSV infections in beef cattle are not equally distributed throughout Sweden and higher-prevalence areas are located in the same southern parts of the country. These results form a basis for further investigations of between-herds dynamics and risk factors for these infections aiming to design effective control strategies. They are also of interest and could be utilized for risk-based approaches in the surveillance of absent or emerging infectious diseases in cattle.

## Competing interests

The authors declare that they have no competing interests.

## Authors' contributions

FB participated in the design of the study, performed the statistical analyses and drafted the manuscript. CB participated in the design of the study and helped to draft the manuscript. SA helped to draft the manuscript. JF participated in the design of the study, contributed to the statistical analysis and helped to draft the manuscript. All authors read and approved the final manuscript.
